# Marinobufagenin inhibits glioma growth through sodium pump *α*1 subunit and ERK signaling‐mediated mitochondrial apoptotic pathway

**DOI:** 10.1002/cam4.1469

**Published:** 2018-03-26

**Authors:** Yu‐Long Lan, Xun Wang, Jia‐Cheng Lou, Jin‐Shan Xing, Shuang Zou, Zhen‐Long Yu, Xiao‐Chi Ma, Hongjin Wang, Bo Zhang

**Affiliations:** ^1^ Department of Neurosurgery The Second Affiliated Hospital of Dalian Medical University Dalian 116023 China; ^2^ Department of Pharmacy Dalian Medical University Dalian 116044 China; ^3^ Department of Physiology Dalian Medical University Dalian 116044 China; ^4^ Department of Neurology The Second Affiliated Hospital of Dalian Medical University Dalian 116023 China

**Keywords:** Glioma, marinobufagenin, mitochondrial apoptotic pathway, therapy

## Abstract

Malignant glioma is one of the most challenging central nervous system diseases to treat and has high rates of recurrence and mortality. Current therapies often fail to control tumor progression or improve patient survival. Marinobufagenin (MBG) is an endogenous mammalian cardiotonic steroid involved in sodium pump inhibition. Currently, various studies have indicated the potential of MBG in cancer treatments; however, the precise mechanisms are poorly understood. The functions of MBG were examined using colony formation, migration, cell cycle, and apoptosis assays in glioma cells. A mitochondrial membrane potential assay was performed to determine the mitochondrial transmembrane potential change, and cytochrome *c* release from mitochondria was assayed by fluorescence microscopy. An immunofluorescence assay was performed, and the nuclear translocation of NF‐*κ*B in glioma cells was confirmed by confocal microscopy. Western blotting and RT‐qPCR were used to detect the protein and gene expression levels, respectively. In addition, transfection experiment of ATP1A1‐siRNA was further carried out to confirm the role of sodium pump *α*1 subunit in the anticancer effect of MBG in human glioma. The apoptosis‐promoting and anti‐inflammatory effects of MBG were further investigated, and the sodium pump *α*1 subunit and the ERK signaling pathway were found to be involved in the anticancer effect of MBG. The in vivo anticancer efficacy of MBG was also tested in xenografts in nude mice. Thus, therapies targeting the ERK signaling‐mediated mitochondrial apoptotic pathways regulated by MBG might represent potential treatments for human glioma, and this study could accelerate the finding of newer therapeutic approaches for malignant glioma treatment.

## Introduction

The bufadienolides are a group of steroid compounds belonging to cardiac glycosides, which is a class of circulating substances 1, 2, 3. Marinobufagenin (MBG), an endogenous mammalian cardiotonic agent 4, 5, is the most commonly used one for drug research. MBG has been implicated in various physiological conditions 6 and appears to be associated with pathophysiological events in animals 7, 8, 9 and humans 10, 11, 12, 13. MBG may be an important endogenous mammalian cardiotonic steroid with an indispensable regulatory effect on a variety of physiological conditions.

Gliomas are by far the most common primary brain tumor in adults and glioblastomas (GBM) represent the ultimate grade of malignancy 14. The overall survival time for patients with glioma is 15 to 18 months after diagnosis 15. Despite advances in the treatment of malignant gliomas, they remain characterized by dismal prognoses 16. Multimodal treatment, including surgical resection combined with chemotherapy and radiation, is the preferential treatment of choice for GBM; however, the highly resistant nature of GBM to chemotherapy and various other conventional therapies have made treatment difficult. Therefore, the development of new treatments is urgently needed.

Mitochondria are the key cellular organelles that play essential roles in various functions. In addition to its well‐established role as a “powerhouse of cell,” mitochondria have recently been accepted as a “motor of cell death” due to their crucial role in programmed cell death or apoptosis 17. Intriguingly, mitochondrial signaling occurs downstream of the ERK signaling pathway 18. In addition, conversely, disordered mitochondria‐induced ROS may also activate ERK (via phosphorylation) possibly through deactivation of certain phosphatases 18. Thus, targeting ERK signaling could affect the mitochondria‐related apoptotic signaling pathway 19. It is known that NF‐*κ*B is a key regulator of the inflammation, and MAPK signaling pathways like those mediated by ERK, JNK, and p38 are indeed critical for NF‐*κ*B translocation and transactivation 20. Specifically, activated ERK (phosphorylated ERK) may facilitate I*κ*B*α* (an upstream regulator of NF‐*κ*B) phosphorylation directly or indirectly, leading to nuclear localization of NF‐*κ*B 18, which is indicative of activation of the inflammatory pathway. Furthermore, NF‐*κ*B also plays a critical role in the regulation of cellular growth and apoptosis 21. Specific blockade of NF‐*κ*B signaling could lead to cell growth inhibition and apoptosis; therefore, NF‐*κ*B appears to be an important target for cancer therapy.

It is known that bufadienolides specifically inhibit sodium pump (Na^+^/K^+^‐ATPase) 22, whose main function unit is *α* subunit. Among all four components of α subunit, α1 subunit is found to be higher expression in human glioma tissues than normal tissue 23 and was generally thought to be a potential target for cancer treatment. As the main component of bufadienolides, MBG was previously found to exert the anticancer effect through *α*1 subunit in this study, and the effect of *α*1 subunit inhibition by MBG on ERK signaling pathway was further determined in this study. Sodium pump *α*1 subunit could be of great significance for the treatment of glioma. Currently, the potential roles of MBG in glioma are poorly understood. A study by Uddin et al. 24 showed that MBG could cause endothelial cell monolayer hyperpermeability by altering apoptotic signaling. The authors demonstrated that MBG could cause disruption of endothelial cell junction integrity, and this supports that MBG increases vascular permeability in endothelial cells for the first time. In addition, Ing et al. 25 found that MBG enhanced the permeability of human brain microvascular endothelial cells (HBMECs). They also found that MBG could downregulate certain genes involved in adhesion in the endothelial monolayers obtained from the human brain. Since tumor endothelial cells have been found to be associated with cancer progression and metastasis 26, 27, the findings mentioned above could indicate a connection between MBG and glioma malignancy. Furthermore, it is known that MBG is involved in the inhibition of the sodium pump 28. Intriguingly, the involvement of the sodium pump in the proliferation and migration of glioma cells has already been determined 29. Thus, these findings further indicate the potential of MBG in cancer treatment and suggest that this warrants more research.

Thus, it was hypothesized that MBG could be of great anticancer effect, and MAPK/ERK signaling and mitochondrial apoptotic signaling pathway might be involved in the cell death induced by MBG in glioma cells. We therefore examined the effects of MBG on cytochrome *c* release, the expression of caspase proteins that are early apoptosis markers, and JC‐1 staining to determine whether MBG causes apoptotic signaling in glioma cells. The ERK inhibitor PD980025 was used to examine the effect of ERK on the mitochondria‐related apoptotic signaling pathway. In addition, the ERK/NF‐*κ*B signaling pathway was also indicated to be involved in the anticancer effect of MBG. The results showed that MBG could induce mitochondrial dysfunction partly through the MAPK/ERK signaling pathway in human glioma cells. Current research could strongly support the anticancer activity of MBG in glioma. This study presents a mechanistic basis for further pharmacokinetic studies or clinical trials to assess the efficacy of MBG as a glioma treatment.

## Materials and Methods

### Antibodies and other materials

The primary antibodies for p‐ERK, p‐JNK, p‐p38, NF‐*κ*B p65, p‐p65, cleaved caspase‐9, cleaved caspase‐3, *β*‐actin, Bcl‐2, Bax, and all the secondary antibodies were purchased from Cell Signaling Technology (Cell Signaling Technology, Inc., Pudong, Shanghai, China). The primary antibodies for ATP1A1, cytochrome C, and NF‐*κ*B p50 were obtained from Santa Cruz Biotechnology (Santa Cruz, CA). Trypsin, fetal bovine serum (FBS), and Dulbecco's modified Eagle's medium (DMEM) were obtained from HyClone Laboratories (HyClone Laboratories Inc., WEST 1800 SOUTH LOGANUT 84321). MBG (BP3644; from Biopurify Phytochemicals, Chengdu, China) was solutioned in DMSO and kept at −20°C. The phosphate‐buffered saline (PBS), protease inhibitor cocktail, and 5‐diphenyltetrazolium bromide (MTT) were purchased from Sigma (St. Louis, MO).

### Cell culture

Human U87MG, U373MG, and U251 glioma cell lines and normal human astrocyte SVG p12 were obtained from ATCC (Manassas, VA). Cells were grown under standard tissue culture conditions (37 °C, 5% CO2) in DMEM containing 10% fetal bovine serum.

### Cell viability assay

MTT assay kit (Roche Diagnostics, Indianapolis, IN) was used to analyze the cell viability. Briefly, glioma cells were seeded at 6 × 10^3^ cells per well in 96‐well plates. Various concentrations of MBG dissolved in DMSO (final concentration, 0.1%) were added in each well. After 48 h incubation, the cell viability was analyzed. The IC_50_ of MBG was determined by interpolation from dose–response curves.

### In vitro migration assay

The cells were seeded in 6‐well plates. The monolayers were scratched with a sterile 200‐*μ*L pipette tip, and then, they were washed with PBS by three times. After that, the cells were cultured in serum‐free medium with indicated doses of MBG and kept in a CO_2_ incubator. The scratch wounds were observed at 0 and 48 h under a Leica DM 14000B microscope fitted with digital camera.

### Colony formation assay

The cells were seeded in 6‐well plates (800 cells per well) and then treated with MBG at different concentrations for 24 h. Then, cells were washed with PBS followed by culture with fresh medium containing 10% FBS. Cells were cultured for another 14 days to allow colony formation. Then, the colonies were calculated after staining with 0.1% crystal violet.

### Apoptosis assay

In brief, after treating with different concentrations of MBG for 24 h, cells were collected and washed three times with PBS. Then, the cells were stained with 5 *μ*L of propidium iodide (Beyotime) and 5 *μ*L of annexin V‐FITC in 500 *μ*L of binding buffer for 10 min in the darkroom according to the manufacturer's instructions. Stained cells were then analyzed by FACS Accuri C6 (Genetimes Technology Inc., Xuhui District, Shanghai, China).

### Cell cycle analysis

U251 cells were incubated with different concentrations of MBG for 24 h. Then, the cells were trypsinized into single cells and collected, followed by washes with PBS, and then suspended in a staining buffer (0.5% Tween‐20, 10 *μ*g/mL propidium iodide, 0.1% RNase in PBS). Then, the stained cells were analyzed by flow cytometer with CellQuest acquisition and analysis software program (Becton Dickinson and Co., San Jose, CA).

### Mitochondrial membrane potentials assay

JC‐1 probe was used to test mitochondrial depolarization in glioma cells. After indicated treatments, cells cultured in six‐well plates were incubated with an equal volume of JC‐1 staining solution (5 *μ*g/mL) at 37°C for 20 min, followed by rinses twice with PBS. Mitochondrial membrane potentials were monitored by counting the relative amounts of dual emissions from mitochondrial JC‐1 monomers or aggregates using an Olympus fluorescent microscope under Argon‐ion 488 nm laser excitation. Mitochondrial depolarization is shown by a trend of decrease in the red/green fluorescence intensity ratio.

### Western blotting analysis

Whole cell extracts were separated via SDS‐PAGE, and the proteins were transferred to PVDF membranes (Millipore, Billerica, MA). The membranes were blocked for 2 h at room temperature in 5% milk and then incubated in primary antibody overnight at 4°C or 4 h at room temperature. The membranes were washed with PBS three times (10 min/wash). Subsequently, the membranes were incubated in secondary antibody for 2 h at room temperature and washed with PBS three times (15 min/wash). Finally, the protein concentration was determined by a BCA protein assay kit.

### ATP1A1 siRNA transfection experiments in vitro

Transfection was performed to downregulate the expression level of *α*1 subunit protein using ATP1A1‐siRNA (RiboBio Co. Ltd., Guangzhou Science City, Guangdong, China) (sequence: GGGCAGUGUUUCAGGCUAA). In vitro experiment, the control‐siRNA and ATP1A1‐siRNA were separately dissolved in Opti‐MEM, followed by mix with transfection reagent‐lipofectamine2000 for 20 min to form siRNA liposomes. Then, the glioma cells were transfected with the siRNA liposomes in antibiotic‐free cell medium. Finally, the expression levels of ATP1A1 and further the cell viabilities were detected after 24 h of transfection in the absence or presence of MBG for an additional 24 h.

### Confocal immunofluorescence

The cells were seeded onto coverslips in a six‐well plate. Then, they were fixed with 4% paraformaldehyde (w/v) for 30 min. Coverslips with glioma cells were then washed with PBS for 15 min and permeabilized with 0.2% (w/v) Triton X‐100 in PBS for 5 min. Then, they were blocked with PBS containing 1% bovine serum albumin (BSA) for 30 min. Coverslips were subsequently incubated with the primary antibodies against cytochrome *c*, p65, and p50 diluted in PBS containing 10% BSA overnight. Coverslips were washed with PBS for three times, and then, fluorescein isothiocyanate‐ and rhodamine‐conjugated secondary antibodies were added in 1% blocking solutions and incubated for 1 h. After three times washes with PBS, coverslips with glioma cells were stained with DAPI (Beyotime). Samples were examined using a Leica DM 14000B confocal microscope.

### Animal studies

BALB/c athymic nude mice (4–6 weeks of age) were used, and each experimental group consisted of 6–10 mice. All animal procedures were carried out in accordance with the guidelines approved by the U.S. National Institutes of Health Guide for the Care and Use of Laboratory Animals. Briefly, U251 cells (2 × 10^6^) were injected subcutaneously into the left and right flanks of the same mice. Tumors were measured perpendicular dimensions using calipers. Volumes were estimated, and tumor specimens were fixed in formalin and embedded in paraffin.

Bioluminescence images for monitoring tumor growth were begun approximately 28 days after tumor implantation using the IVIS Spectrum system (Caliper, Xenogen, Alameda, CA). The tumor‐bearing mice were anesthetized (isoflurane/O_2_ in an induction chamber; isoflurane from Baxter International Inc., Deerfield, IL), and a solution of d‐luciferin (120 mg/kg in PBS in a total volume of 80 mL; Biosynthesis, Naperville, IL) was administered s.c. in the neck region. Anesthesia was maintained with isoflurane (2%) in oxygen (1 dm^3^/min). Five min after luciferin injection, an array of various exposure times (1, 5, 30, 60 sec) was applied for image acquisition. Data were quantified with the Living Imaging software using absolute photon counts (photons/sec) in a region of interest (ROI) that was manually drawn to outline the bioluminescence image signals.

On day 30, all experimental mice were terminated with ether anesthesia. Total weight of the tumors in each mouse was measured. To further analyze the expression of p65 NF‐*κ*B, the tumor tissues were freshly fixed with 10% neutral formalin, then desiccated, followed by embedded in paraffin. 4 *μ*m sections were stained with hematoxylin and eosin, p‐p65 NF‐*κ*B antibody (1:150), and examined under a light microscope.

### Statistical analysis

Statistical analysis was exerted using SPSS20.0 statistical software (SPSS Inc., Chicago, IL). Student's *t*‐test analysis was used to examine the difference of means among different groups. The values were presented as the mean ± stand. *P* < 0.05 was considered statistically significant.

## Results

### MBG inhibited U251, U87MG, and U373MG cell proliferation and suppressed colony formation and migration of glioma cells

**Figure 1:**
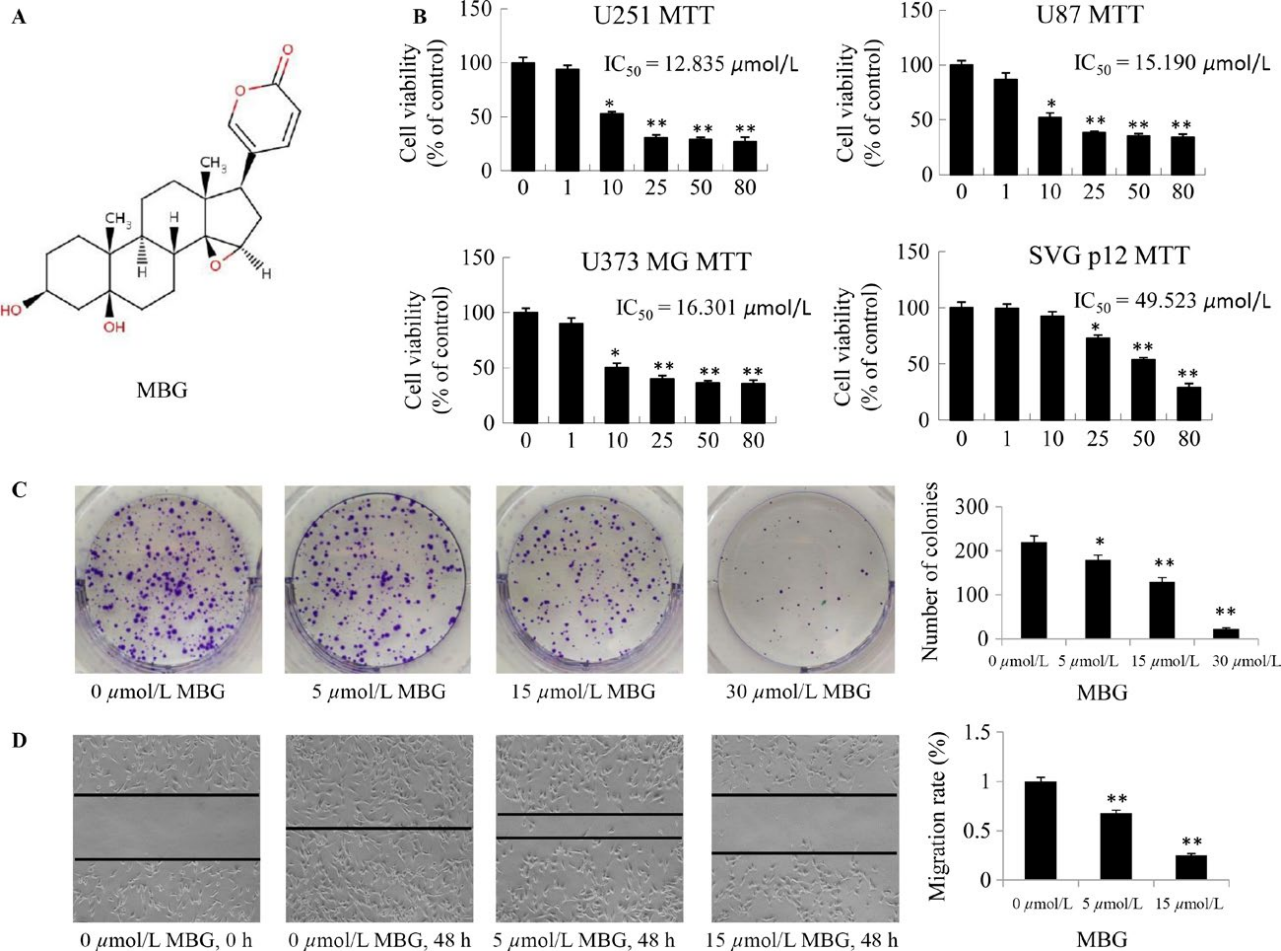
MBG inhibited cell viability and changed cell morphology. (A) Chemical structure of MBG. Molecular weight: 400.508 g/mol. Human U87MG, U373MG, and U251 glioma cells and normal human astrocytes (SVG p12) were treated with MBG at the indicated doses under normal culture conditions. (B) The cell viability was determined by an MTT assay at 48 h after treatment. (C) Colony formation in U251 tumor cells was also analyzed, and the colony formation rate was calculated. (D) Cell migration was analyzed using a wound‐healing assay. The data are presented as the mean ± SD of three separate experiments. (*P < 0.05, **P < 0.01, significant differences between MBG treatment groups and DMSO vehicle control groups).

The chemical structure of MBG is shown in Figure 1A. The anticancer effects of MBG were detected in U251, U87MG, and U373MG cells. Based on an MTT assay, MBG was found to inhibit the proliferation of U251, U87MG, and U373MG cells in a concentration‐dependent manner, while MBG had less effect on the growth of the normal human astrocyte cell line SVG p12 (Fig. 1B). The data showed that the IC_50_ values of MBG for U251, U87MG, and U373MG cells were 12.835, 15.190, and 16.301 *μ*mol/L, respectively (Fig. 1B). Consistent with inhibition of cell proliferation, MBG also significantly inhibited colony formation (Fig. 1C). A wound‐healing assay further indicated the inhibitory effect of MBG on tumor cell mobility in U251 cells. It could be shown that for U251 cells, the MBG‐treated cells failed to occupy the scraped space through migration, while the migrating cells in the control group occupied the wounding space (Fig. 1D). These results suggest that MBG could be of well properties for suppressing cell proliferation, migration, and colony formation of human glioma cells.

### MBG induced apoptosis and altered the cell cycle

**Figure 2:**
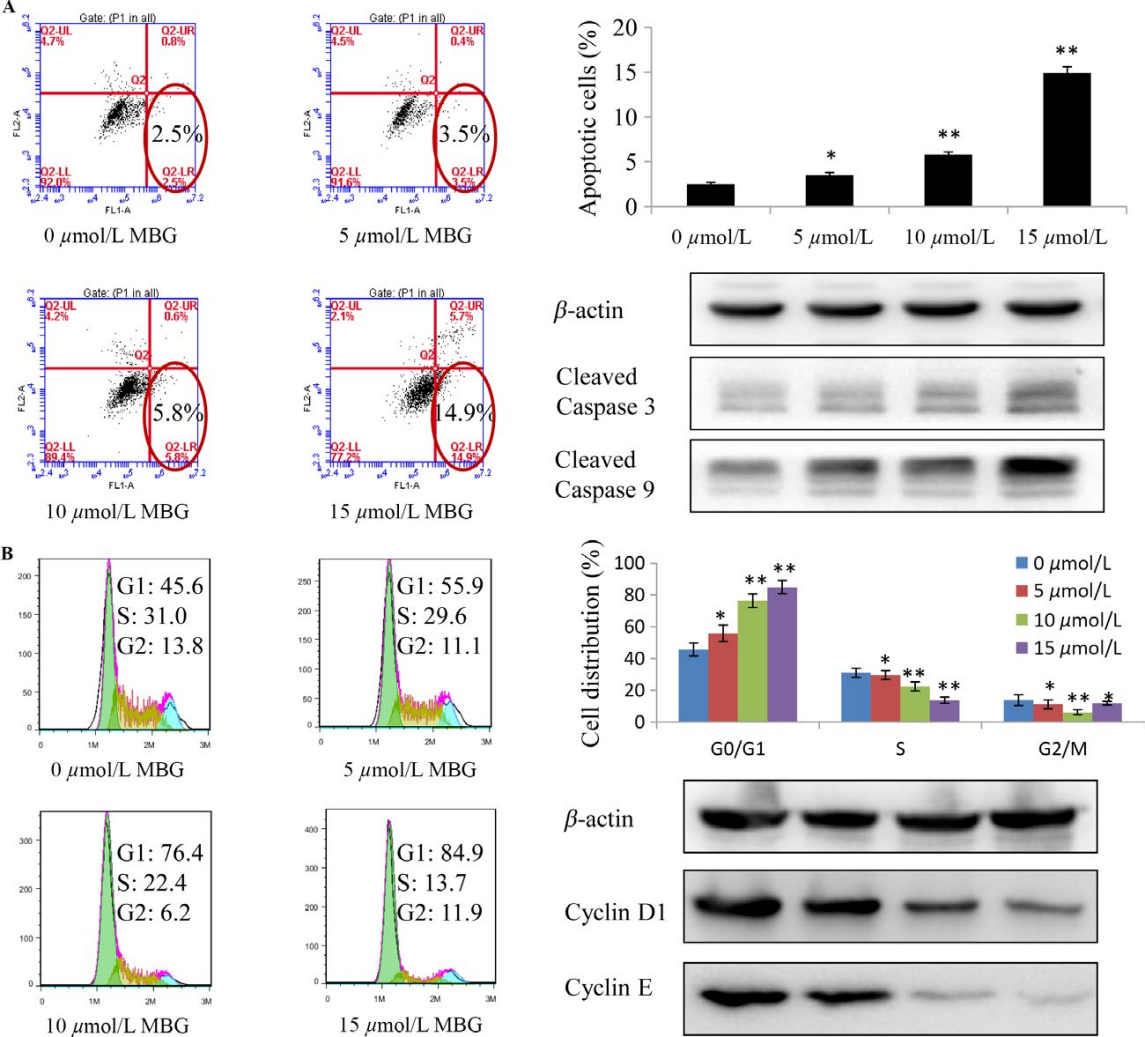
MBG induced apoptosis and altered cell cycle progression. (A–B) Human U251 cells were treated with MBG at the indicated doses. (A) Cells were treated with MBG at the indicated times. Apoptosis was determined by FACS analysis, and the levels of cleaved caspase‐3/9 in U251 cells were analyzed by Western blotting. (B) MBG induces G1 phase arrest in U251 cells. Flow cytometric analysis was performed to further examine the effect of MBG on the proliferation of GBM cells as a result of altering cell cycle progression. Cells were treated with 5, 10, and 15 *μ*mol/L MBG for 48 h, and the DNA content was analyzed by flow cytometry. The percentage of cells in the G1, S, and G2/M phases of the cell cycle are shown. In addition, Western blotting of protein extracts obtained from U251 cells that were treated with 0, 5, 10, or 15 *μ*mol/L MBG was also performed. Total protein extracts were prepared after treatment for 48 h and analyzed with antibodies to Cyclin D1 and Cyclin E. Significant differences from the control were indicated as follows: *P < 0.05 and **P < 0.01.

MBG‐induced apoptosis of U251 cells was observed. Condensed chromatin was observed by FACS analysis in MBG‐treated U251 cells (Fig. 2A), and the MBG‐induced apoptosis was increased in a dose‐dependent manner (Fig. 2A). To confirm the effect of MBG on apoptosis, we detected the expression of three key pro‐apoptotic proteins (caspase‐3 and caspase‐9) in U251 cells by Western blotting. The MBG‐treated group had markedly increased expression levels of the cleaved caspase‐9 and caspase‐3 proteins (Fig. 2A). To understand the inhibitory mechanism of MBG on cell growth, cell cycle analysis was performed (Fig. 2B). The U251 cells were exposed to the indicated concentrations of MBG for 24 h. As shown in Figure 2B, cells were arrested in the G1 phase, the percentage of cells in the S phase decreased, while the G2/M phase did not change significantly. In addition, the protein expression related to the cell cycle further confirmed these results (Fig. 2B).

### MBG treatment increases apoptosis by inducing mitochondrial dysfunction

**Figure 3:**
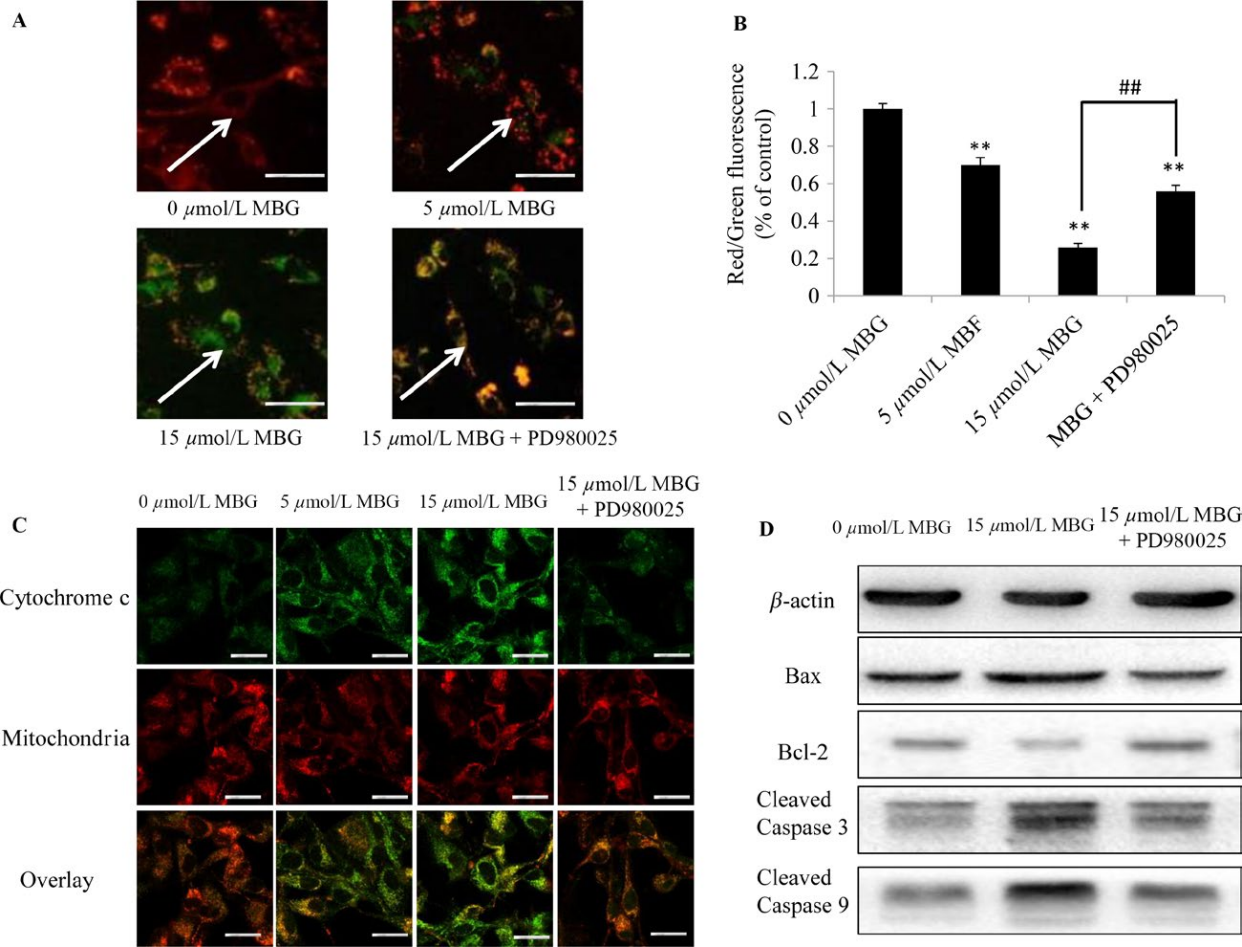
MBG treatment induced mitochondrial dysfunction. (A–B) U251 cells were exposed to the indicated doses of MBG for 24 h. Changes in the mitochondrial membrane potential were determined by JC‐1 staining and fluorescence microscopy. We observed that this change could be partially reversed by the ERK signaling inhibitor PD980025. Bar = 50 *μ*m. *n* = 3. (B) A quantitative analysis of the shift of mitochondrial orange‐red fluorescence to green fluorescence among the groups (red/green fluorescence ratio) was conducted. (C) Cytochrome *c* release from the mitochondria of the cells was determined by fluorescence microscopy after treatment with 0, 5, or 15 *μ*mol/L MBG and 15 *μ*mol/L MBG plus the ERK signaling inhibitor PD980025 for 48 h. Bar = 25 *μ*m. *n* = 3. (D) Bax, Bcl‐2 and cleaved caspase‐3/9 protein levels in U251 cells were analyzed using Western blotting. All values are denoted as the mean ± SD from ten independent photographs from each group. **P < 0.01 compared with control cells cultured in complete medium; ^##^ *P* < 0.01 compared with the indicated treatment group.

In this study, JC‐1, which is a fluorescent probe, was used to test the mitochondrial membrane potential. Significantly, increased dose‐dependent cell death was inversely correlated with decreased mitochondrial membrane potential in MBG‐induced glioma cells based on the results of the JC‐1 assay. Treatment with 5 and 15 *μ*mol/L MBG for 24 h could cause a decrease in the mitochondrial membrane potential, as detected by enhanced green intensity and reduced red intensity of JC‐1. However, intriguingly, the use of PD980025 (an ERK inhibitor) could reverse the decrease in the mitochondrial membrane potential (Fig. 3A). Subsequently, the ratio of red and green fluorescence intensity was determined using a fluorescence microplate reader, which indicated a dose‐ and time‐dependent decrease (Fig. 3B).

Additionally, in the activation of apoptosis, the release of cytochrome *c* from the mitochondria into the cytosol is a critical step. Next, using immunofluorescence imaging (IF) analysis, we monitored the changes in the subcellular localization of cytochrome *c* in U251 cells to determine whether MBG could induce the release of cytochrome *c*. Treatment with MBG (5 *μ*mol/L or 15 *μ*mol/L) markedly triggered the release of cytochrome *c* from the mitochondria into the cytosol (Fig. 3C). The results showed that MBG significantly increased cytochrome *c* release in glioma cell cultures, while PD980025 markedly inhibited MBG‐induced cytochrome *c* release (Fig. 3C), suggesting that ERK signaling was involved in the MBG‐induced cytochrome *c* release. To further confirm the underlying mechanism of ERK signaling in glioma cells in response to MBG induction, immunoblotting was performed to analyze the apoptosis‐related proteins in glioma cells in the presence or absence of PD980025. The results showed that MBG could induce ERK phosphorylation, while the presence of PD980025 remarkably inhibited the activation of phosphorylated ERK (Fig. 3D). These data clearly suggest that the activation of the ERK signaling pathway plays a major role in the response to MBG in human glioma cells.

### MBG inhibited NF‐*κ*B translocation from the cytoplasm to the nucleus

**Figure 4:**
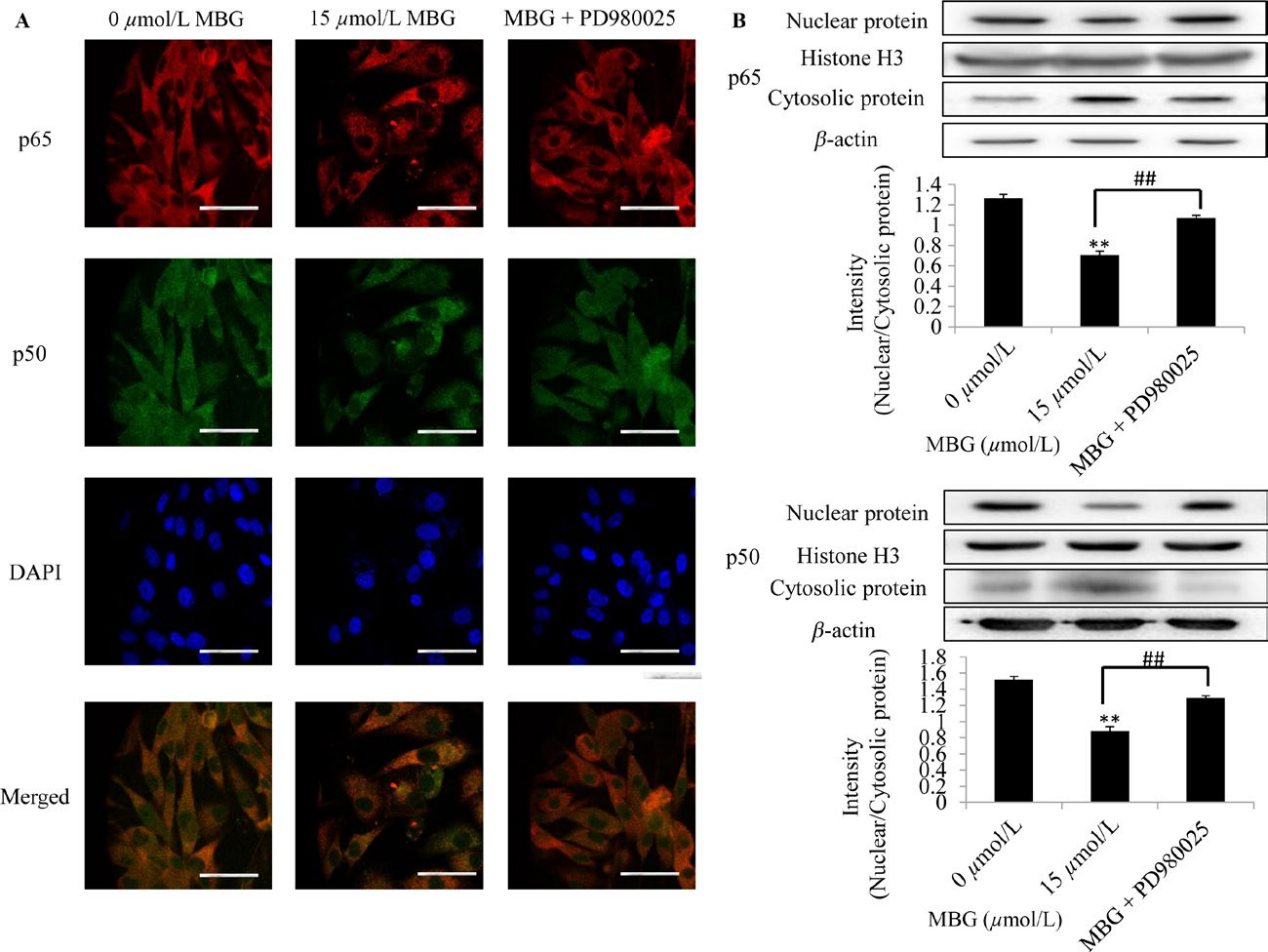
MBG inhibited NF‐*κ*B translocation from the cytoplasm to the nucleus. (A) At 24 h after treatment, the subcellular localization of p50 and p65 and the colocalization of p65 with p50 were examined by confocal microscopy. MBG was found to inhibit NF‐*κ*B translocation from the cytoplasm to the nucleus, and such translocation could be partially inhibited by the ERK signaling inhibitor PD980025. Bar = 50 *μ*m. *n* = 3. (B) Nuclear and cytosolic NF‐*κ*B expression levels were further analyzed by Western blotting. *P < 0.05 and **P < 0.01 compared with the control cultures; ^##^ *P* < 0.01 compared with indicated treatment group.

NF‐*κ*B is known to be an important transcription factor that plays a critical role in the control of cellular growth and apoptosis 21. An immunofluorescence assay was performed, and the nuclear localization of NF‐*κ*B and the colocalization of p65 and p50 in U251 cells were confirmed by confocal microscopy (Fig. 4A). MBG treatment inhibited NF‐*κ*B translocation from the cytoplasm to the nucleus, while the use of PD980025 reversed this inhibition. In addition, constitutive translocation of NF‐*κ*B p50/p65 to the cell nucleus was detected in U251 cells by Western blotting (Fig. 4B). These findings indicated that, compared with the DMSO control, treatment with MBG markedly inhibited the translocation of the NF‐*κ*B p65/p50 proteins from the cytoplasm to the nucleus (Fig. 4A and B). The results indicate that MBG might inhibit U251 cell proliferation via inhibiting NF‐*κ*B translocation from the cytoplasm to the nucleus.

### MBG inhibited proinflammatory mediators and suppressed phosphorylation of ERK MAPKs

**Figure 5:**
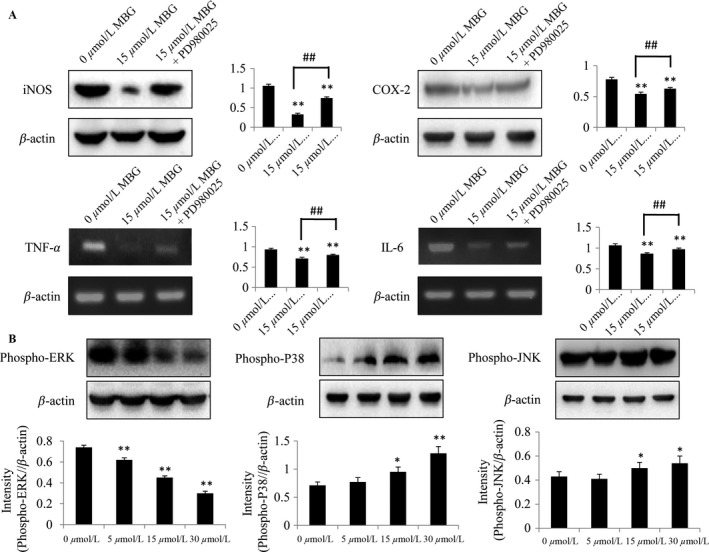
MBG could exert anti‐inflammatory and anticancer effect through the ERK signaling pathway. (A) Effect of MBG on the expression of proinflammatory mediators in glioma cells. Human U251 cells were treated with MBG at the indicated doses. At 48 h after treatment, expression levels of IL‐6 and TNF‐*α* gene were analyzed by RT‐qPCR, while expression levels of iNOS and COX‐2 protein were analyzed by Western blotting in U251 cells, respectively. (B) MBG suppressed the phosphorylation of ERK MAPKs in U251 cells. Western blotting and quantitative analysis further revealed that MBG specifically targets p‐ERK, while MBG does not target p‐p38 or p‐JNK. *n* = 3. *P < 0.05 and **P < 0.01 compared with the control cultures; ^##^ *P* < 0.01 compared with indicated treatment group.

It is known that NF‐*κ*B regulates the expression of various proinflammatory mediators, such as inducible nitric oxide synthase (iNOS), cyclooxygenase‐2 (COX‐2), interleukin 6 (IL‐6), and tumor necrosis factor *α* (TNF‐*α*). To further confirm the effect of MBG on the ERK/NF‐*κ*B signaling pathway, we further assessed the expression of these proinflammatory mediators. At 48 h after treatment, expression levels of IL‐6 and TNF‐*α* gene were analyzed by RT‐qPCR, and expression levels of iNOS and COX‐2 protein were determined by Western blotting (Fig. 5A) in U251 cells, respectively. It was observed that MBG reduced the protein expression of iNOS and COX‐2, and decreased the gene expression of TNF‐*α* and IL‐6 in a dose‐dependent manner. All these confirmed that in the treatment of MBG could reduce expressions of the proinflammatory mediators significantly.

MAPKs are generally accepted as upstream regulators of NF‐*κ*B. Therefore, we aimed to explore the possible upstream regulators targeting MAPKs that could influence NF‐*κ*B activation. In mammalian cells, isoforms of the ERK MAPKs are the best‐studied members of the family 19. As inflammation induces hyperphosphorylation and activation of various components of the MAPK family, namely ERK, JNK, and p38, the phosphorylation status of these MAPKs was investigated by Western blotting (Fig. 5B). The results indicated that MBG suppressed phosphorylation of ERK MAPKs in U251 cells, while such suppressing effect was not applicable for JNK and p38 MAPKs. Together, these results indicate that MBG inhibited the inflammatory response in part through regulation of MAPK signaling, by targeting p‐ERK in glioma.

### Sodium pump *α*1 subunit could mediate the ERK‐targeted anticancer effect of MBG

**Figure 6:**
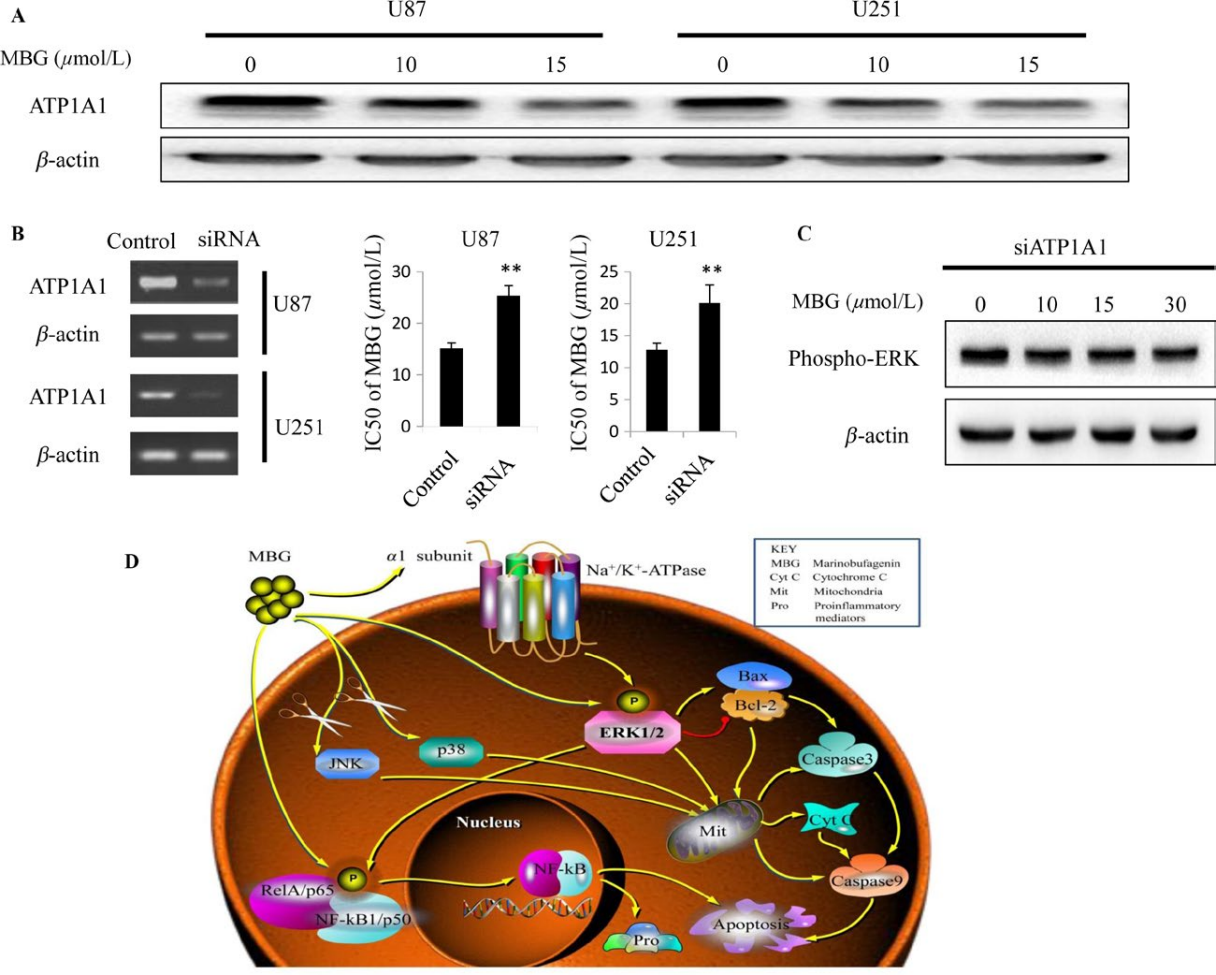
Sodium pump *α*1 subunit could mediate the ERK‐targeted anticancer effect of MBG. (A) MBG inhibited ATP1A1 protein expression in human glioma cells. U87MG and U251 cells were treated with MBG, respectively, after 48 h, cells were harvested for ATP1A1 level measurement by Western blotting. (B) Knocking down ATP1A1 markedly changed the IC_50_ values of MBG for glioma cells. U87MG cells were treated with si‐ATP1A1 for 5 h to knockdown ATP1A1 expression, then incubated in normal DMEM medium supplemented with 15% fetal bovine serum for another 24 h. After that, cell lines were seeded at 6 × 10^3^ cells/well in 96‐well plates, and cells were allowed to adhere for overnight. Then, cells were treated with MBG at indicated doses. MTT assay was exerted to determine cell viability, thus to determine whether ATP1A1 knockdown could hinder the anticancer effect of MBG in human glioma cells. (C) MBG‐induced inhibition of ERK phosphorylation could be impeded by ATP1A1 knockdown. Western blotting was used to determine the levels of ERK phosphorylation in U87MG cells treated with MBG under ATP1A1 knocking down. (D) Schematic diagram of an apoptotic cell death induced by MBG via the ERK signaling pathway. All values are denoted as the mean ± SD. *n* = 3. **P < 0.01 compared with the control cultures.

We determined whether MBG could induce ATP1A1 reduction. U87MG and U251 cells were treated with MBG, respectively, for 48 h before cell harvesting. As shown in Figure 6A, MBG treatment markedly inhibited ATP1A1 expression levels in U87MG and U251 cells. We then measured the effect of ATP1A1 knockdown on the anticancer effect of MBG using U87MG cells. Cells were treated with si‐ATP1A1 to knockdown ATP1A1 expression (Fig. 6B) and treated with MBG at various doses, and then, we measured the IC_50_ values of MBG for U87MG and U251 cells. As shown in Figure 6B, ATP1A1 knockdown markedly changed the IC_50_ values of MBG for glioma cells. More importantly, MBG‐induced inhibition of ERK phosphorylation could be impeded by ATP1A1 knockdown (Fig. 6C). As expected, ATP1A1 knockdown could in part block the anticancer effect of MBG. Taken together, these results indicate that MBG could inhibit glioma growth via binding to ATP1A1.

Besides, the anti‐inflammatory mechanisms of MBG regarding its effect on the ERK/NF‐*κ*B signaling pathway and the ERK signaling‐mediated mitochondrial apoptotic pathway are represented graphically in Figure 6D. The molecular activity of dopamine will be our next area of investigation.

### MBG caused decreased tumorigenicity in vivo

**Figure 7:**
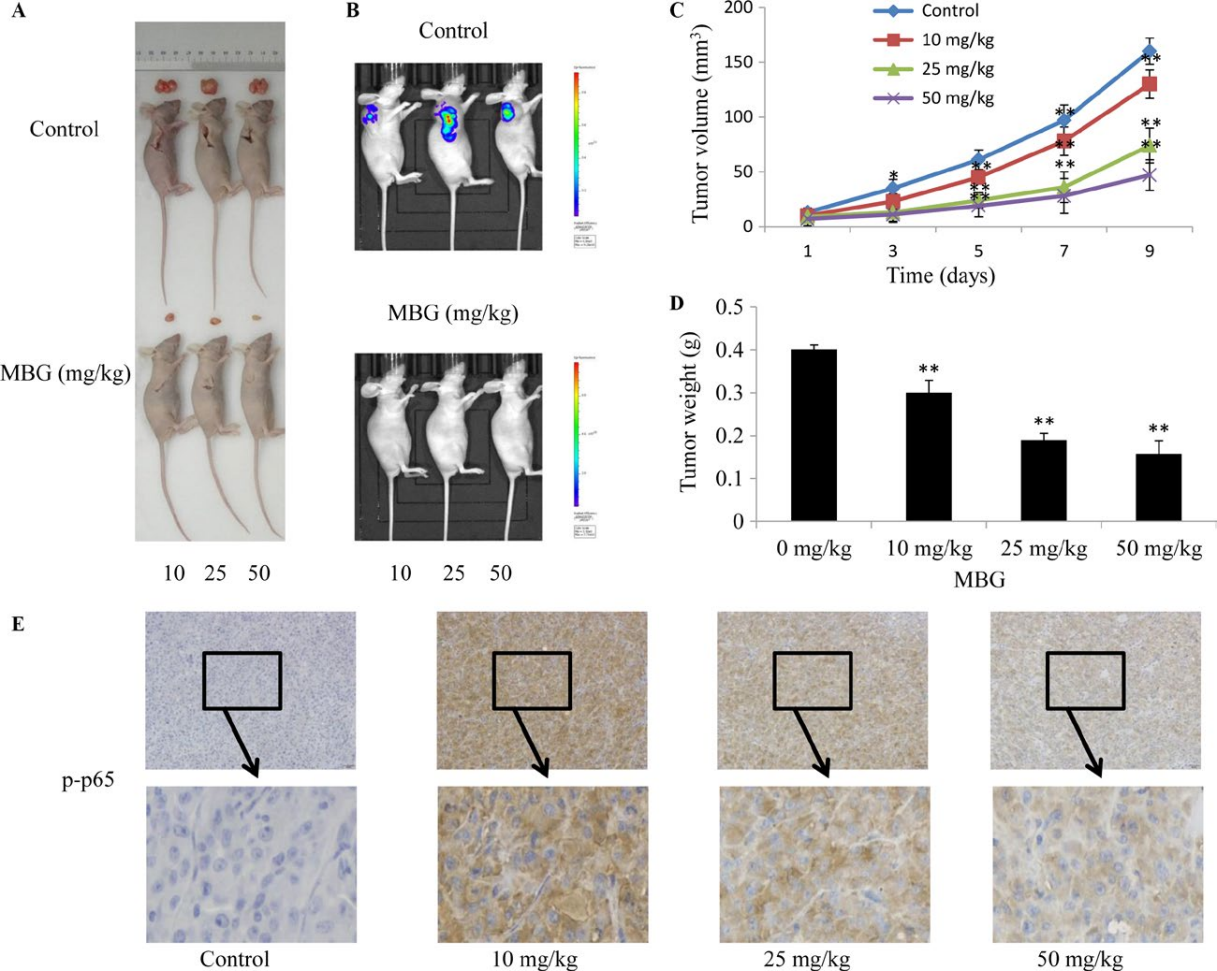
The in vivo anticancer efficacy of MBG was analyzed in xenografts in nude mice. The rats of the U251 glioma subcutaneous model received MBG (0, 10, 25, and 50 mg/kg) or vehicle by intraperitoneal administration once every 2 days for 9 days (A). Mice were also imaged 28 days after implantation by representative bioluminescence images (B). The rats were euthanized 24 h after completion of treatment, and tumor volumes and weights were measured (C–D). (E) Immunohistochemical analysis of p‐p65 protein expression in the tumor samples. All values are denoted as the mean ± SD. *n* = 3. **P < 0.01 compared with the control cultures.

To detect the inhibitory effect of MBG on U251 cells in vivo, we performed elementary tumor xenografts in nude mice. Mice‐bearing subcutaneous tumors were treated with therapy 9 days after tumor cell injection. During the 9 days, one mouse from each group was sacrificed. Mice were divided into three treatment groups (Fig. 7A). In addition, in vivo imaging detection was performed, and representative bioluminescence images clearly showed that mice in the MBG treatment groups had significantly longer tumor growth delays than mice in the control groups (Fig. 7B). After administration of MBG in the mice with U251‐xenografts for 9 days, both the tumor volumes (Fig. 7C) and the tumor weights (Fig. 7D) of the treated mice decreased significantly when compared with those of the control mice. In addition, an immunohistochemical staining assay was used to test the expression of p‐p65, which is the activated form of p65. Compared with the vehicle group, the expression levels of p‐p65 were significantly decreased in vivo in the MBG treatment group (Fig. 7E). These results indicated that dopamine could inhibit the growth of xenografted human glioma cells.

## Discussion

Toad venom is an anti‐inflammatory drug traditionally used for the treatment of various types of inflammation 30, 31. MBG, which is the major active ingredient of toad venom, exhibited significant antitumor activities, with good drug characteristics 30. Meanwhile, it has been found that toad venom extracts or bufadienolides could also be of anti‐inflammatory effects through inhibiting the proliferation of human T lymphocytes 31, but currently little is known about the effects of other constituents of toad venom, and only a few studies have investigated the biological activities of MBG regarding its anticancer properties.

In the present study, we examined the anticancer effects of MBG through testing its effect on cytochrome c release, the expression of caspase proteins that are early apoptosis markers, and JC‐1 staining to determine whether MBG causes apoptotic signaling in glioma cells. In addition, the ERK/NF‐*κ*B signaling pathway also appeared to be involved in the anticancer effect of MBG. Taken together, these results indicated that MBG could effectively inhibit glioma growth through ERK‐mediated mitochondrial apoptotic pathways. In our study, considering that U251 and U87MG cells are more applicable for morphological observation and that U251 cells are better able to form xenografts in nude mice, most experiments in vitro were performed in U251 cells study the molecular mechanisms by which MBG suppresses glioma growth.

The transformation of the mitochondrial membrane potential could convert the transformation of the membrane permeability 32 by a mechanism involving the cytochrome *c* release and the activation of the caspase‐3/9 cascade 33. The increased release of cytochrome *c* and activation of caspase3/9 signaling were observed in MBG‐treated U251 cells in the current study, suggesting that a mitochondria‐related signaling pathway was involved in the MBG‐induced apoptosis of glioma cells. Besides, mitochondrial signaling occurs downstream of the ERK signaling pathway. Indeed in the present study, the inhibition of ERK signaling using PD980025 restored Bax protein (a pro‐apoptotic protein) expression and Bcl‐2 protein (an anti‐apoptotic protein) expression and suppressed cytochrome *c* release and caspase‐3/9 activation in U251 cells. Together with evidence that disordered mitochondria‐induced ROS activates ERK signaling 18 and MBG inhibits the ERK signaling pathway, all these suggested that the mitochondria signaling pathway was mediated by ERK signaling in glioma cells in response to the use of MBG.

NF‐*κ*B is maintained in an inactive state in the cytoplasm. In our study, we confirmed that MBG inhibited the translocation of NF‐*κ*B from the cytosol to the nucleus. Furthermore, MBG has also been found to inhibit the expressions of proinflammatory mediators, including iNOS, COX‐2, TNF‐*α*, and IL‐6. In addition, the present results showed that MBG specifically targets p‐ERK, while not the p‐p38 or p‐JNK. Studies regarding the mechanisms by which the structural identity of MBG specifically recognizes and inhibits target MAPKs are underway. As mentioned above, we showed that MBG inhibited inflammation via suppression of NF‐*κ*B and p‐ERK MAPKs in glioma cells.

Furthermore, sodium pump *α*1 subunit could be of great significance for the treatment of glioma, and MBG was further confirmed to be able to exert the anticancer effect through *α*1 subunit in the current study, and the effect of *α*1 subunit inhibition by MBG on ERK signaling pathway was also determined. Recent studies have indicated that high expression of sodium pump on the membrane surface of mammalian cells is closely related to the occurrence, development, and malignancy of cancer, and it could be of important roles in the ion balance, signal transmission, energy metabolism, and morphological structure of cancer cells 34. The current study indicated that *α*1 subunit in the membrane of cancer cells could be the new target of anticancer effect of MBG, and significantly, MBG could be of greater therapeutic significance for glioma with high expression of *α*1 subunit. Many reports have confirmed that the expression of *α*1 subunit is higher in glioma cell lines and tissue samples of patients than that in normal tissue 23. Thus, the study regarding the expression of *α*1 subunit in GBM might be of great significance for the treatment and prognostic evaluation of this intractable disease, and this would be our future direction.

In conclusion, we attempted to uncover the mechanisms underlying the anticancer effects of MBG. The results revealed a mechanism of cell death‐regulated signaling pathways in the mitochondria of glioma cells in response to MBG (Fig. 6D). Mechanistically, MBG activates ERK/NF‐*κ*B signaling, activating the caspase signaling cascade through accelerating the release of cytochrome *c*, thus sequentially inducing apoptosis. We found that MBG suppresses glioma growth, both in vivo and in vitro. These findings provide strong evidence supporting the use of MBG as a novel anticancer agent in glioma treatment.

## Conflict of Interest

The authors declare no competing financial interests.
